# The potential of oviduct tags and fine‐scale acoustic telemetry to reveal the timing and location of spawning in Arctic salmonids (*Salvelinus* spp.)

**DOI:** 10.1111/jfb.15951

**Published:** 2024-10-07

**Authors:** Véronique Dubos, Les N. Harris, Richard Ekpakohak, Brendan K. Malley, Matthew J. H. Gilbert, Nathan B. Furey, Jean‐Sébastien Moore

**Affiliations:** ^1^ Institut de Biologie Intégrative et des Systèmes, Département de Biologie Université Laval Québec Quebec Canada; ^2^ Fisheries and Oceans Canada, Arctic and Aquatic Research Division Winnipeg Manitoba Canada; ^3^ Elder and Cambridge Bay Resident Cambridge Bay Nunavut Canada; ^4^ Department of Biology University of New Brunswick Saint John New Brunswick Canada; ^5^ Institute of Arctic Biology and Department of Biology and Wildlife University of Alaska Fairbanks Alaska USA; ^6^ Department of Biological Sciences University of New Hampshire Durham New Hampshire USA

**Keywords:** Arctic char, fine‐scale positioning, lake trout, nest guarding, reproduction, egg incubation, spawning habitat, spawning site, VPS

## Abstract

Identifying and characterizing spawning locations are paramount for the protection of critical fish habitats but can be challenging, particularly in remote locations. Using the underexplored oviduct‐tagging technique, we aimed to identify the timing and location of spawning for wild Arctic char (*Salvelinus alpinus*) and lake trout (*Salvelinus namaycush*) in two high‐Arctic lakes in Nunavut. Specifically, Innovasea V7 acoustic telemetry transmitters were inserted into the oviducts of 13 Arctic char and 4 lake trout, and the timing and location of tag expulsion were determined using a fine‐scale positioning system. Twenty Arctic char and 20 lake trout were also tagged with abdominal V16 transmitters, and 10 of them were paired with the oviduct tags, to further study the behavior of individual fish during the spawning season. Oviduct tags from four Arctic char and one lake trout could be used to assess the timing and location of spawning. Spawning anadromous Arctic char drastically reduced their activity and remained proximate to their presumed spawning location immediately before and for months after spawning. In contrast, a non‐anadromous (i.e., freshwater resident) Arctic char and a lake trout showed little to no reduction in activity around presumed spawning events. Because of the highlighted sedentary behavior of inferred spawning anadromous Arctic char implanted with both abdominal and oviduct tags, we could also infer potential spawning based on the behavior of individuals equipped only with abdominal tags. Spawning areas identified via telemetry also aligned well with Inuit knowledge of those lakes. This is the first field study to use acoustic oviduct and abdominal tags coupled with a fine‐scale positioning system. Despite a limited success rate of ejection, the study reveals the strong potential of the method to study spawning habitat and timing, particularly in remote areas.

## 1 INTRODUCTION

Spawning is a critical life‐history event, and fish in particular exhibit a remarkable diversity of reproductive tactics (Knapp & Neff, 2008). Successful spawning is vital for sustaining and rebuilding populations (Taylor et al., 2019), and having a clear understanding of the reproductive ecology of aquatic organisms has implications for understanding individual fitness and ultimately population and species persistence (Brommer et al., 2002). Moreover, identifying the spatiotemporal characteristics of spawning events is essential for site‐specific habitat protection (Cooke et al., 2023; Dey et al., 2021; Taylor et al., 2019) and to understand how spawning activities may change in space and/or time under altered climatic or environmental conditions (Kanamori et al., 2019; Petitgas et al., 2013; Rogers & Dougherty, 2019). Detailed information on the spatiotemporal aspects of spawning in many fishes, however, is frequently lacking, particularly for Arctic species (Bilous et al., 2022).

Conventional approaches for observing and documenting spawning behavior in fishes typically involve the direct observations or capture of spawning individuals in their spawning habitats. However, most of these approaches still require a priori information on spawning behavior (e.g., timing and location) and environmental requirements, as well as habitats that allow for direct observations, monitoring, or the capture of spawning individuals. In addition to the need for prior information, these methods often require intensive efforts, rendering their application costly and in some cases can even adversely impact spawning individuals, their spawning activities, or their spawning habitats (Uhlmann & Broadhurst, 2015). Traditional ecological knowledge (Bliss et al., 2023), passive acoustic monitoring (Straight et al., 2014), and remote sensing (Ponsioen et al., 2023) have also been used to describe spawning behavior and habitats. Acoustic telemetry is a powerful tool for tracking the movement and behavior of aquatic animals (Hussey et al., 2015; Kraft et al., 2023; Marsden et al., 2021; Matley et al., 2022), and it has been specifically applied to the study of the reproductive ecology of many fishes (Douglas et al., 2009; Gutowsky et al., 2020). Despite its potential, most studies using acoustic telemetry to investigate spawning have focused on sites and species where the timing and location of spawning activities were already known (Binder et al., 2018; Gatch et al., 2023; Marsden et al., 2016). Although the timing and location can sometimes be inferred based on behavior (e.g., reduced home ranges and levels of activity), a few studies have used acoustic telemetry to identify exactly when and where spawning takes place when these are unknown (Gardner & Höök, 2024; Skovrind et al., 2013). The advent of fine‐scale acoustic telemetry positioning systems (Kraft et al., 2023; Lennox et al., 2023) has improved our ability to study spawning activity and behavior with higher precision (Binder et al., 2014, 2018; Dean et al., 2014).

Significant technological advances have facilitated the development of biotelemetry transmitters small enough to be inserted into the oviducts of mature females in a variety of fish species. These transmitters can then be expelled with the eggs during spawning, allowing to infer the exact timing and location of spawning events (Binder et al., 2014). This method was first tested with mini radio transmitters inserted into the oviducts of northern pike (*Esox lucius*) and muskellunge (*Esox masquinongy*) (Pierce, 2004; Pierce et al., 2007). Acoustic telemetry tags inserted in the oviducts of European perch (*Perca fluviatilis*), paired with manual tracking, allowed expelled tags to be located with an accuracy of 10–125 m (Skovrind et al., 2013). Combining oviduct insertion methods with fine‐scale positional telemetry would obviate manual tracking requirements and allow for the identification of specific spawning sites with high precision (Espinoza et al., 2011). Indeed, Binder et al. (2014) tested the feasibility of using oviduct‐inserted acoustic transmitters in lake trout in a laboratory setting and suggested that they could be used with positional telemetry to estimate the timing and location of spawning. To the best of our knowledge, however, this method has not been tested in the field, especially not in remote locations such as the Arctic where such approaches would be even more advantageous due to logistical constraints.

Arctic char (*Salvelinus alpinus*) and lake trout (*Salvelinus namaycush*) play key roles in northern ecosystems and are the cornerstone of commercial, recreational, and subsistence fisheries throughout the northern extent of their range (Harris, Yurkowski, et al., 2022; Kissinger et al., 2020; Marin et al., 2017; Watts et al., 2017). Both species have also been central to Inuit culture, food security, and health for centuries (Harris, Moore, et al., 2022; Norman & Friesen, 2010) and remain among the most important subsistence resources in the Canadian Arctic (Priest & Usher, 2004). Despite the importance of these species across Arctic Canada, our knowledge of their reproductive ecology is lacking in many areas, largely due to the remoteness of sampling locations and because these areas are often inaccessible during spawning in fall and early winter when ice conditions are not safe. Anadromous Arctic char are known to spawn in a wide variety of lacustrine and fluvial environments (Johnson, 1980), whereas lake trout generally spawn in lacustrine habitats characterized by rocky, clean substrates ranging in size from gravels to boulders (Callaghan et al., 2016; Marsden et al., 1995, 2016). In the central Canadian Arctic, spawning in Arctic char occurs from mid‐September to October (Johnson, 1980). The timing of spawning in lake trout is highly variable and can range from August to December, and depths where spawning takes place also vary widely across its range (Binder et al., 2021; Muir et al., 2021). Only a handful of studies, however, have focused on specifically identifying spawning habitats of these salmonids in Arctic regions (but see Callaghan et al., 2016; Dubos, May, et al., 2023), with some studies being informed by Inuit knowledge (Cunjak et al., 1986; Harwood & Babaluk, 2014). Here we used oviduct‐inserted acoustic transmitters paired with conventional surgically implanted abdominal acoustic transmitters to study Arctic char and lake trout in two Arctic lakes where fine‐scale acoustic positioning systems were installed. Our aim was to determine the utility of this approach in (a) identifying the precise timing and location of spawning and (b) assessing behavior before, during, and after spawning.

## 2 METHODS

### 2.1 Study area and VEMCO positioning system acoustic telemetry array

**FIGURE 1:**
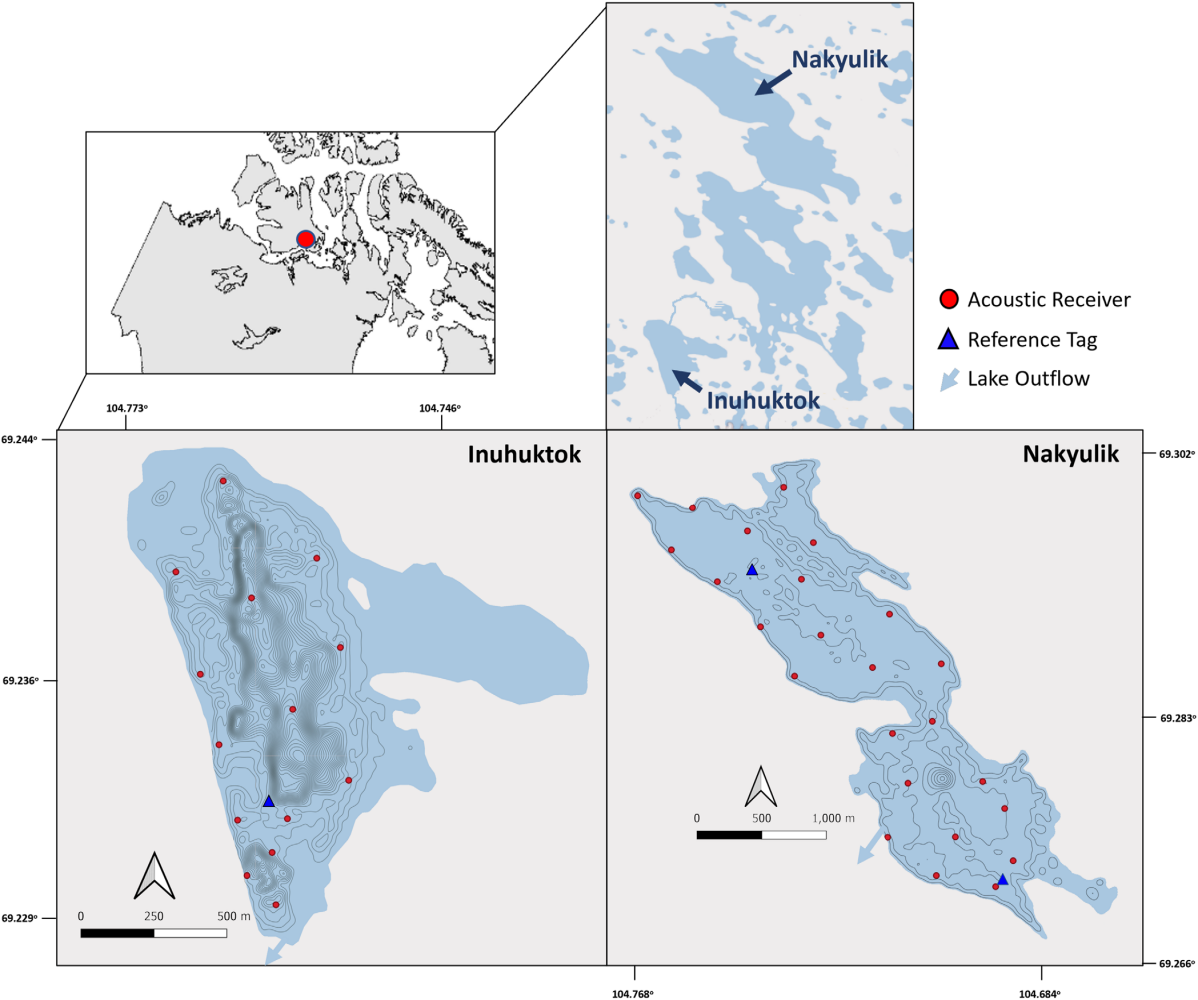
Study area and location of acoustic receivers in the two lakes studied: Inuhuktok and Nakyulik.

This study was conducted in two High Arctic lakes, Inuhuktok and Nakyulik, near the community of Iqaluktuuttiaq (Cambridge Bay), Victoria Island, Canada (Figure 1). These lakes were identified as known Arctic char and lake trout spawning lakes through local knowledge (R. Ekpakohak, personal observation). Both lakes are within the Greiner Lake watershed that drains Freshwater Creek, the mouth of which is 2 km from the community. Inuhuktok is 20 m above sea level with a surface area of 1.11 km^2^ and a maximum depth of 31 m (Rautio et al., 2022). The shallow eastern branch of the lake freezes to the bottom and is not accessible to mature char and trout in winter. Nakyulik is located 8 km upstream from Inuhuktok in the Greiner watershed. It is 26 m above sea level with a surface area of 16 km^2^ and a maximum depth of 10.5 m (Rautio et al., 2022). The two lakes are connected during the ice‐free period. Neither lake thermally stratifies, and both contain lake trout and resident and anadromous Arctic char. Other fishes such as lake whitefish (*Coregonus clupeaformis*), cisco (*Coregnus* spp.), and nine‐spined stickleback (*Pungitius pungitius*) also occur in the watershed.

We deployed the VEMCO positioning system (VPS), an acoustic telemetry positioning system, which requires an array of acoustic receivers with overlapping detection radii to be able to record simultaneous detections of an acoustic tag on three or more receivers for triangulation (Orrell & Hussey, 2022). This system uses a time of difference of arrival algorithm to relate the differences in time among tag detections along with the sound of water to estimate a high‐resolution position of the tag (Smith, 2013). Between August 20, 2018, and August 27, 2018, 14 receivers (Innovasea VR2AR, 69 kHz) were deployed in Inuhuktok and 24 receivers were deployed in Nakyulik (Figure 1). Each receiver was placed ~1–2 m off the bottom. Station depth ranged from 4.0 to 24.0 m in Inuhuktok and 3.2–7.1 m in Nakyulik. Each receiver also recorded temperature (±0.5°C) at 60‐min intervals, and internal synchronization tags (required for internal clock synchronization) were set to “high” power. The recorded temperatures of three and four receivers in Inuhuktok and Nakyulik, respectively, were averaged to obtain mean lake temperatures. Reference tags (Innovasea VX, 69 kHz) were also deployed in each lake (one in Inuhuktok and two in Nakyulik) to assess VPS performance over time. The receivers were retrieved between July 24 and 27, 2019.

### 2.2 Acoustic transmitter implantation

#### 2.2.1 Abdominal tags

Lake trout and Arctic char in Inuhuktok and Nakyulik were tagged during August 19–29, 2018. Individual fish (*n* total = 40, *n* Arctic char = 20, *n* lake trout = 20; Table S1) were fitted with intracoelomic (abdominal) acoustic transmitters (V16‐4x, diameter: 16 mm, length: 68 mm, weight: 24 g; Vemco Ltd., Bedford, NS), half of which were equipped with pressure (depth) and temperature sensors (V16TP‐4x, diameter: 16 mm, length: 71 mm, weight: 26 g, accuracy: ±0.5°C and ±1.7 m; Vemco Ltd.). V16 acoustic transmitters were programmed with a nominal delay of 350 s for 153 days and 600 s for 212 days, a programme that was repeated for the battery life of the transmitter (V16 = 1825 days, V16TP = 3650 days).

#### 2.2.2 Oviduct tags

A subset of females from both species (*n* total = 17 between Arctic char [AC] and lake trout [LC]; *n*_AC, Inuhuktok_ = 3, *n*_AC, Nakyulik_ = 10, *n*_LT, Nakyulik_ = 4) were also implanted with smaller acoustic transmitters (V7‐4x, diameter: 7 mm, length: 21.5 mm, weight: 1.8 g, estimated battery life: 539 days; Vemco Ltd.) in their oviduct, following procedures described in Binder et al. (2014, tab. 1). The V7 oviduct implanted transmitters were programmed with a nominal delay of 240 s. These tags were active throughout the presumed spawning period (August 1, 2018, to November 1, 2018), shut off for winter, and reactivated the following year, June 1, 2019. This programming was chosen to extend the battery life to confirm that tags assumed to be expelled during spawning were indeed stationary the following spring. One of the V7 oviduct tags (4947, fish 27529, Inuhuktok) was inadvertently programmed to stop later than November 1, 2018, and was last detected on November 27, 2018.

#### 2.2.3 Combined abdominal and oviduct tags

In Inuhuktok, three Arctic char were implanted with both V16 (abdominal) and V7 (oviduct) transmitters. In Nakyulik, four Arctic char and three lake trout were implanted with both V16 and V7 transmitters (Table S1); we focus much of our analyses on these seven fish. In this lake, six additional Arctic char and one lake trout were implanted only with a V7 transmitter in the oviduct (i.e., they did not have a V16 concurrently implanted in the abdominal cavity). This complex study design and the small sample sizes among tags deployed between species and lakes reflect limitations related to complex field logistics and conditions in these High Arctic environments; access to both these species and specific lakes was limited during our study period, preventing larger deployments for a given species or system. The mean tag burden, including with combined tags, was 0.90% ± 0.41% of the fish weight, varying from 0.06% to 1.56%.

#### 2.2.4 Tag implantation

Fish were captured via continuously monitored gillnets and angling. Captured fish were immediately placed in a holding cage within the lake for subsequent assessment of tagging suitability (typically >6 h). Fish deemed suitable for tagging were anaesthetized in an MS‐222 (75 ppm) and sodium bicarbonate buffer solution (1:2). The implantation of acoustic transmitters followed standard surgical procedures previously used on these species in the region (Harris et al., 2020; Harris, Yurkowski, et al., 2022; Moore et al., 2016). Briefly, fish fork length and weight were measured, and anaesthetized fish were placed in a padded V‐shaped cradle and provided with a constant stream of a weaker maintenance anesthetic (50 mg/L MS‐222) over the gills. An incision was made on the ventral side of the fish to insert the transmitter and then closed using simple interrupted stitches (2‐0 curved needle, coated Vicryl suture, Ethicon J459H). A small sample of pectoral fin was collected to sex the fish using genetic analysis (Yano et al., 2013). Upon completion of the surgery, fish were placed in a post‐op tub of fresh water and observed until they fully recovered; then they were released back into the lake. Transmitters for oviduct insertion were gently inserted through the oviduct and pushed ~5 cm deep using a 10‐cm‐long piece of sterilized 6.5‐mm‐diameter Tygon tubing. After insertion, fish were returned to the post‐surgery tub of fresh water to recover from anesthesia as described earlier. The principles and procedure were approved by the CPAUL (*Comité de protection des animaux de l'université Laval*, 2017066‐1) and DFO animal care committee (AUP FWI‐ACC‐2017‐019).

### 2.3 Filtering estimated animal positions

The estimated horizontal positions of tags through time were calculated using Innovasea from across all detections. We acknowledge that the VPS is a proprietary system over which the authors have limited control regarding how positions are derived from the raw detections. Other approaches, like the open‐source software YAPS (Baktoft et al., 2017), provide alternative systems for positioning. We excluded a few false detections identified by tag IDs that did not match our transmitters. One Arctic char (V16: 6231, V7: 4954) was detected during the whole study period but did not exhibit any movement and presumably died shortly after being tagged; we did not include this fish in any further analyses. Each position estimated by the VPS is assigned a horizontal position error (HPE) estimate. HPE is a unitless measure of the potential precision of a position based largely on the geometry of the receivers used to estimate a position and the location of the transmitter position relative to these receivers (Smith, 2013). HPE can then be related to measured horizontal position error in meters (HPEm) for stationary synchronization and reference tags, for which the “true” positions are known (Smith, 2013), to attempt to relate HPE to potential horizontal errors and identify thresholds for filtering (Roy et al., 2014). This point‐by‐point approach for filtering VPS positions is commonly used, but particularly in reflective environments a track‐oriented approach such as YAPS may perform better (Vergeynst et al., 2020). We calculated the mean, median, and 95th percentile of HPEm of synchronization and reference tags for each one‐unit bin of HPE (e.g., HPE between 9 and 10, 10 and 11). From these values, we decided to use thresholds of HPE <30 in Inuhuktok and HPE <14 in Nakyulik for retaining fish positions. For Inuhuktok, this resulted in a median HPEm of 7.6 m, a mean HPEm of 9.3 m, and a 95th percentile of HPEm of 22.0 m; for Nakyulik the median, mean, and 95th percentiles of HPEm of 4.8, 6.7, and 17.6 m, respectively, among retained synchronization and reference tag positions. In Inuhuktok, 85.5% of the Arctic char positions and 83.4% of the lake trout positions were retained. In Nakyulik, 86.4% of Arctic char and 82.3% of lake trout positions were retained.

### 2.4 Inferred spawning behavior

The VPS data were recorded from the end of August 2018 to the end of July 2019. However, the current analysis focused on data representative of the pre‐ and postspawning activities, that is from August 25, 2018, to December 31, 2018; data were collected after this date but were not included in our analyses here. All fish that interrupted their horizontal movements around the spawning and incubation period resumed their travel later in winter or spring, with the exception of 6193, which remained stationary. This fish ceased to be detected from December 22, 2018 to July 11, 2019, when it was detected again at the same location until July 23, 2019, and is thus presumed dead. Given our focus on the potential utility of oviduct tags to identify spawning areas and timing for Arctic char and lake trout, we visualized the paired locations (oviduct tag and intracoelomic tag in the same fish) via both simple maps (latitude and longitude) as well as assessed the latitude (northing) of both tags through time. These allowed us to (a) visually assess if oviduct tags were colocated with animal tags, (b) assess if oviduct tags were potentially shed from the body, and (c) determine where and when oviduct tag shedding occurred to potentially identify spawning sites. The potential spawning events were inferred from oviduct tags in two ways. First, spawning was inferred when oviduct tags ceased to be detected during the spawning season. In that case, we presumed the tags were buried in the substrate as observed by Binder et al. (2014) for 78% of oviduct tags shed by lake trout during spawning. Second, spawning events were inferred when paired oviduct and intracoelomic tags became separated during the spawning season. In addition, the spawning of some anadromous Arctic char without oviduct tags could be inferred based on similarity in behavior (sedentary for at least 3 weeks) observed in Arctic char that successfully shed oviduct tags during the plausible spawning season. For this, the plausible spawning season window was considered to be from September 25, as has previously been noted for the earliest known spawning timing in the region (Johnson, 1980) and as observed by local Inuit fishers, to 3 weeks after the last oviduct tag shed (November 2, 2018, i.e., November 23).

### 2.5 Observed location of spawning sites

Coauthor R. Ekpakohak, an experienced Inuit fisher and elder, has observed spawning Arctic char in Inuhuktok and Nakyulik. He witnessed them while the ice cover was in place, yet thin enough to allow good visibility of the fish and their nests. He mapped the observed site locations independently from the study results on June 20, 2023, and validated the mapped locations on April 11, 2024. These spawning site locations are presented to support the results of our present analyses (Figure 5).

### 2.6 Estimating hatch timing

To further assess the plausibility of the timing of presumed spawning events identified from telemetry, we estimated a potential hatching date for each successful oviduct tag. Median hatch dates were predicted based on the accumulated thermal units (ATU) after each spawning event, because temperature is a central environmental driver of developmental rate in salmonids, including in lake trout and Arctic char (Dwyer, 1987; Koops & Tallman, 2004). Freshwater temperatures never fell below 0°C, so ATUs above 0°C were simply calculated as the sum of the daily mean temperature for each day after a presumed spawning event. Lake trout hatching was estimated to occur at 352 ATU after spawning as determined by Dwyer (1987) at 1.8°C, which was similar to the observed mean lake temperature during incubation in the present study (1.6°C). Arctic char hatching was predicted using the formula provided by Koops and Tallman (2004). This formula describes the ATU required for hatching to occur as a function of the incubation temperature and was based on data collected from 14 studies on the developmental rates of Arctic char (Koops & Tallman, 2004). Hatch dates were predicted for both species for all presumed spawning events to determine how differences in spawn timing could affect hatch dates.

## 3 RESULTS

During the study period, Arctic char and lake trout were detected 1,241,012 times in Inuhuktok and 1,424,893 times in Nakyulik (including oviduct tag detections). From these detections, Innovasea estimated 136,468 positions (V7 oviduct and V16 abdominal tags) from the VPS in Inuhuktok Lake and 182,008 positions in Nakyulik Lake, after filtering using HPE. The VPS consistently generated animal positions through the study period while tags were actively transmitting (Figures S1–S4). Among individual fish, the number of VPS positions from V7 oviduct tags ranged from 1 to 3380 (mean: 2131 positions, SD = 1732 positions in Inuhuktok; and mean: 713 positions, SD = 925 positions in Nakyulik), and the number of positions from V16 abdominal tags ranged from 9 to 12,393 (mean: 7932 positions, SD = 3118 positions in Inuhuktok; and mean: 7198 positions, SD = 3148 positions in Nakyulik).

### 3.1 Detection of spawning events

**FIGURE 2:**
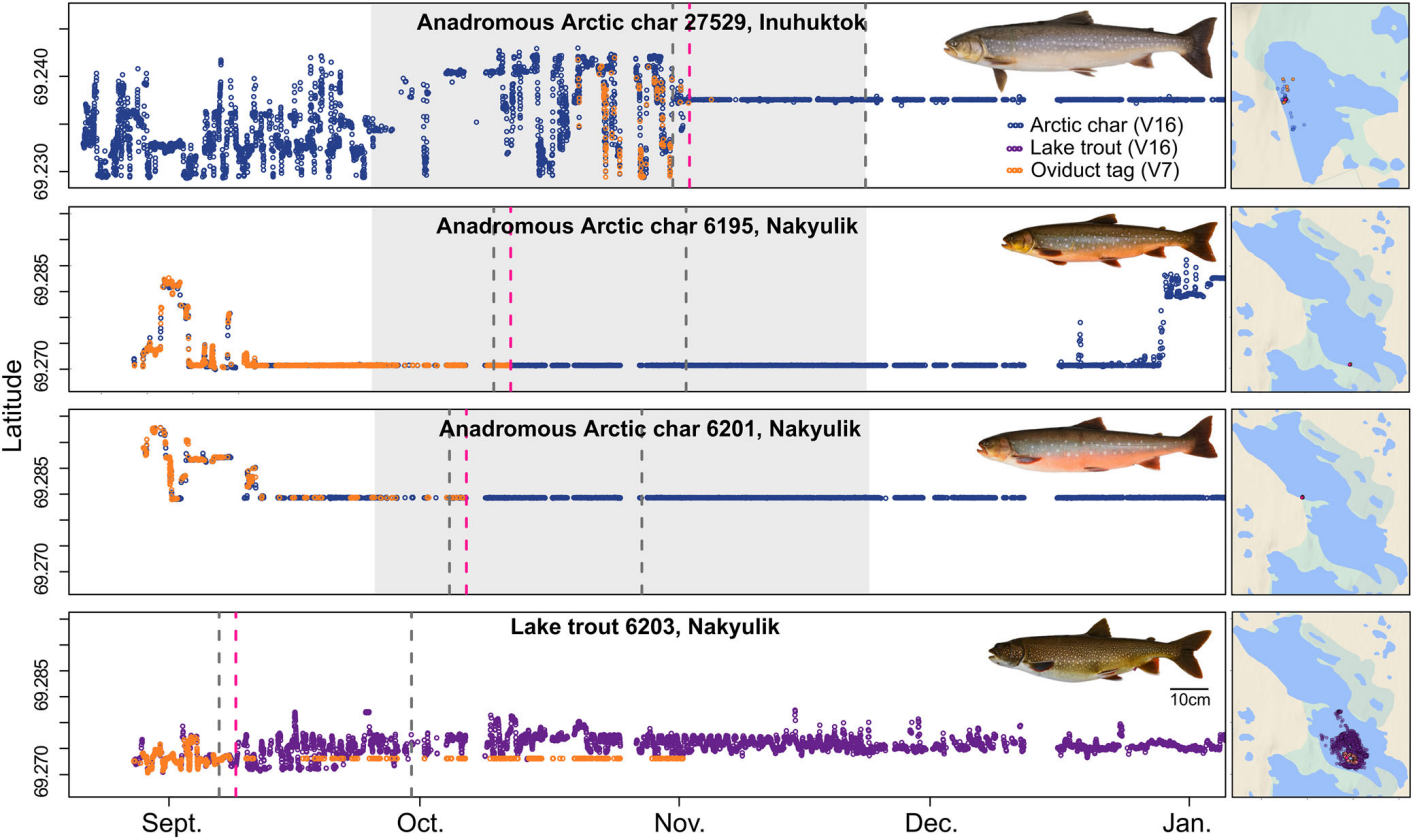
Movements in 2018 of the tagged Arctic char (*Salvelinus alpinus*, top three panels) and lake trout (*Salvelinus namaycush*, bottom panel) that are presumed spawners shown as latitudes of positions through time (left panels) and 2D (two‐dimensional) positions over a period starting 2 days prior to the inferred spawning date and continuing for 3 weeks after, in each lake (right map panels). The pink dashed vertical line identifies the tag ejection and presumed spawning date due to separation of the oviduct tag and abdominal tag or due to ceasing of detections from the oviduct tag (presumably due to ejection into the substrate). The gray vertical dashed lines show the range of dates illustrated on each 2D map (2 days before and 3 weeks after spawning) to describe horizontal movement patterns around the presumed spawning period and to highlight potential spawning areas identified via oviduct tag ejection. The gray shaded area shows what we considered the plausible Arctic char spawning season. Arctic char 27529 and 6201 resumed horizontal movements in spring 2019 (not shown).

Of the 17 deployed, 7 oviduct tags were shed and inferred as spawning, namely 6 of 13 for Arctic char (~46.2%) and 1 of 4 for lake trout (25%) (Table 1; Figures S5–S7). Two Arctic char in Nakyulik, not equipped with an abdominal tag but only an oviduct tag (4948 and 4953), were presumed to have spawned as their oviduct tags ceased to be detected, presumably after being buried in the substrate. However, without an abdominal tag, the spawning timing, nor their migratory status (resident or anadromous), could not be assessed. Three of the six Arctic char that shed oviduct tags during spawning were confirmed to be anadromous, as they left the lake in June (via telemetry data), with other tagged anadromous Arctic char that were detected in the marine environment the same or subsequent years. These anadromous Arctic char were also equipped with abdominal tags (27529 in Inuhuktok, and 6195 and 6201 in Nakyulik; Table 1), and therefore, their spawning behavior could be described (Figure 2). One of the five Arctic char presumed to have shed their oviduct tags during a spawning event was a resident Arctic char of Inuhuktok (27530). However, this fish lost its oviduct tag at an unknown date while the tag was not transmitting, that is, after November 1, 2018 (Figure S8). Residency of some Arctic char (including 27530) was confirmed by regular detections within the array year‐round and the absence of summer migration for subsequent years (L. Harris, unpublished data). The lake trout in Nakyulik (6203) that shed its oviduct tag also had an abdominal tag (Figure 2; Table 1).

**TABLE 1:** Fish with oviduct tags shed during inferred spawning.

Lake	Fish and V7 tag IDs	Comments
Inuhuktok	27529 (V16 abdominal); 4947 (V7 oviduct)	Anadromous Arctic char; spawned on November 2, 2018.
Inuhuktok	27530 (V16 abdominal); 4949 (V7 oviduct)	Resident Arctic char; spawning timing unknown; oviduct tag ejected after November 1, 2018.
Nakyulik	4948 (V7 oviduct)	Arctic char; undetermined migratory status; no paired abdominal tag; spawning location known; postspawning behavior unknown; spawning timing unknown; oviduct tag ejected after November 1, 2018.
Nakyulik	4953 (V7 oviduct)	Arctic char; undetermined migratory status; postspawning behavior unknown; spawned on October 12, 2018.
Nakyulik	6195 (V16 TP abdominal); 4957 (V7 oviduct)	Anadromous Arctic char; spawned on October 11, 2018.
Nakyulik	6201 (V16 TP abdominal); 4956 (V7 oviduct)	Anadromous Arctic char; spawned on October 6, 2018.
Nakyulik	6203 (V16 TP abdominal); 4964 (V7 oviduct)	Lake trout; spawned on September 8, 2018

*Note:* More information on all tagged fish is presented in Table S1, including fish fork length, weight, and sex.

Spawning events could not be inferred for 10 oviduct tags (~59% of those deployed), including 7 Arctic char and 3 lake trout. Three oviduct tags (*n* = 2 in Arctic char and *n* = 1 in lake trout, all in Nakyulik) were presumably not ejected during the study period, as the positions of the oviduct tag and the abdominal tag remained superposed throughout the fall and in the following spring, when the V7 resumed transmitting. The other tags (*n* = 6 Arctic char and *n* = 2 lake trout) were ejected a few hours to 3 days after tagging, which we considered too premature for a spawning event. Statistical power was low, but fork length was similar between fish that successfully ejected their oviduct tags (606 ± 72 mm, mean ± SD) and those that had prematurely expelled their tags (612 ± 103 mm).

### 3.2 Spawning behavior and timing

The abdominal tags paired with the oviduct tags provided detailed information on behavior before and after the spawning period (Figure 2). After initial travel within the lake, anadromous Arctic char significantly reduced their horizontal movements and became largely sedentary for varying durations before the presumed spawning date (e.g., a few hours for 27529 in Inuhuktok to nearly a month for 6195 and 6201 in Nakyulik; Figure 2). After their presumed spawning events (i.e., ejection of the oviduct tag), the three anadromous char remained at the same location for several months, which we inferred was their spawning site (Figure 2). The dual‐tagged lake trout (6203) slightly decreased its horizontal movements 2 days before presumed spawning in the vicinity of the spawning area. Horizontal movements resumed shortly after spawning to activity levels similar to those observed before spawning (Figure 2).

The first fish to shed its oviduct tag in a presumed spawning event was the lake trout in Nakyulik (6203), on September 8, 2018, before ice formation (Figure 2). The inferred spawning for the two Arctic char in Nakyulik (6195 and 6201) was approximately a month later and 5 days apart, on October 6, 2018, and October 11, 2018. Two other Arctic char were inferred to have spawned in Nakyulik based only on V7 tag detection (4948 and 4953; no abdominal tag; Figure S6; Table S1). Due to the absence of a paired abdominal tag, their postspawning behavior could not be assessed, but presumed spawning occurred on October 12 2018, for 4953. For the only anadromous Inuhuktok Arctic char (27529; Table 1) for which we inferred spawning based on an oviduct tag, spawning was estimated to be ~3 weeks later than both Nakyulik Arctic char, on November 2, 2018. Given differences in tag programming, spawning could be inferred for this individual after November 1, 2018.

### 3.3 Vertical habitat use and experienced water temperatures

**FIGURE 3:**
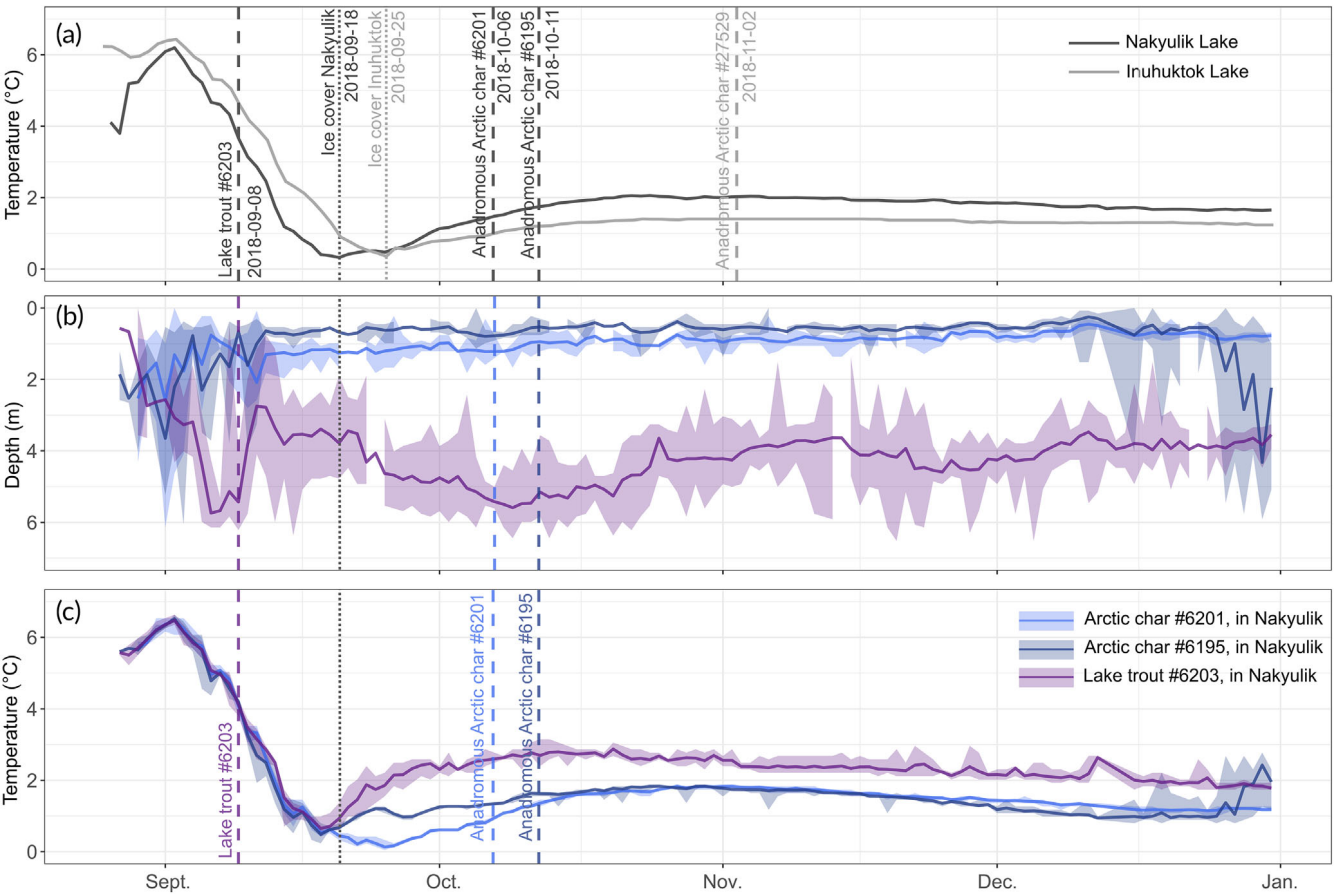
(a) Mean water temperature recorded in each lake in 2018. The vertical dashed lines correspond to inferred spawning dates of the fish that shed oviduct tags. (b) Daily mean depth and (c) daily mean temperature used by the spawning fish equipped with oviduct tags and sensor tags in Nakyulik only (no spawning fish in Inuhuktok were equipped with sensor tags). The shaded areas show the minimum and maximum daily temperature recorded.

For fish with both abdominal sensor tags and oviduct tags we were able to describe the water depths and water temperatures experienced before, during, and after spawning (Figure 3). The lake trout (6203) spawned at 4°C while the water temperature in Nakyulik was steadily decreasing (Figure 3a). The ice cover formation, which occurred at the minimum water temperature in September, happened a week earlier in Nakyulik than in Inuhuktok, respectively, on September 18, 2018, and September 25, 2018. Once the ice cover had formed, the water temperature at Nakyulik rewarmed faster than at Inuhuktok. The two identified spawning events of Arctic char in Nakyulik happened during the winter increase in lake temperature. The first spawning of Arctic char recorded in Nakyulik occurred at 1.4°C. This same temperature was reached 18 days later in Inuhuktok. The Arctic char inferred to have spawned in Inuhuktok also did so at the same temperature (1.4°C), which was the maximum reached over winter (Figure 3c).

The two oviduct‐tagged spawning Arctic char in Nakyulik (6195 and 6201) occupied a constant shallow depth in the littoral zone for several months, starting more than 3 weeks before the spawning date; the mean depths occupied by these fish during October and November were 0.57 ± 0.11 m for Arctic char 6195 and 0.92 ± 0.15 m for Arctic char 6201 (Figure 3b). During this period, both Arctic char occupied the upper and coolest part of the water column and experienced a mean temperature of 1.55C and 1.57°C, respectively (median: 1.64°C). In comparison, the tagged lake trout used deeper water (mean = 5.23 m) during spawning time. After the ice cover had formed (September 18, 2018) and throughout the study period, the lake trout experienced an average temperature of 0.9°C warmer than the two monitored spawning Arctic char, due to the greater depths used and the weak but existing winter reverse thermal stratification of the lake.

### 3.4 Predicted hatching dates after spawning

**FIGURE 4:**
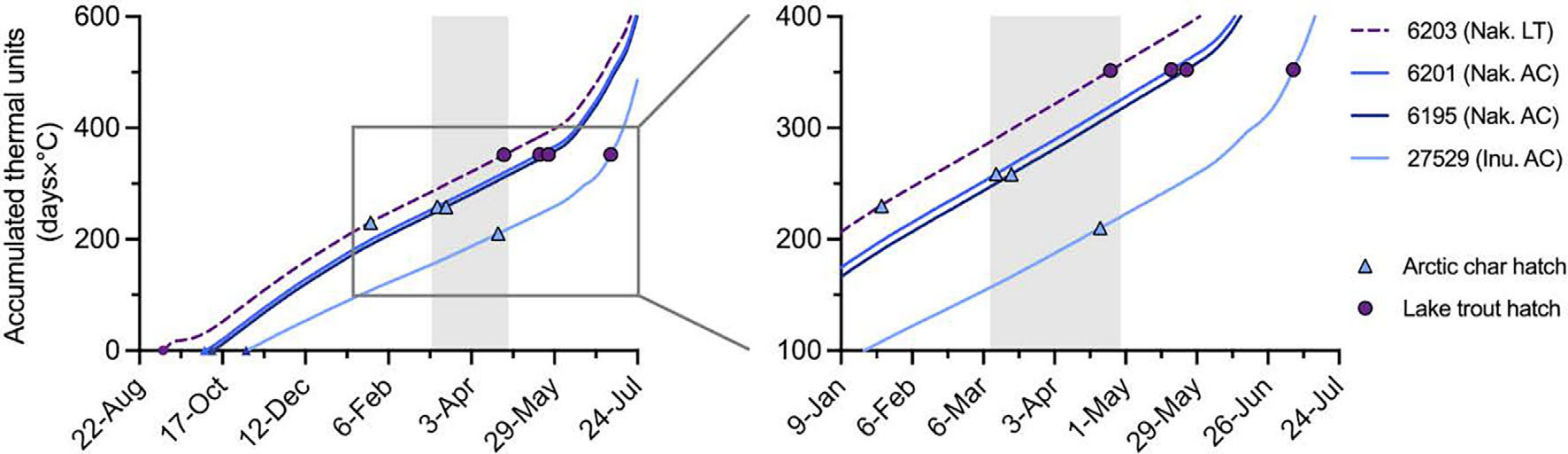
Accumulated thermal units (ATU) in two Arctic lakes, Nakyulik (Nak.) and Inuhuktok (Inu.), after inferred spawning events (*n* = 4), with median hatching dates simulated for lake trout (*Salvelinus namaycush*) (LT; purple, dashed line, and circles) and Arctic char (*Salvelinus alpinus*) (AC; blue, triangles) embryos after each event. Predictions were made for both species at all spawning dates to show the potential species‐specific effect of spawning date on hatch timing. Shading indicates the range of actual predicted median hatching dates corresponding with the presumed spawning of each individual. Fish ID in the legend is ordered by presumed spawning date.

After the lake trout spawning event (6203), the average lake temperature during the estimated incubation period (i.e., up to 352 ATUs) was 1.56°C, resulting in a predicted incubation duration of 225.6 days and a median hatch date of April 25, 2019 (Figure 4). For Arctic char, the average lake temperatures during incubation were 1.65 and 1.64°C in Nakyulik (6201 and 6195, respectively) and 1.24°C in Inuhuktok (27529). These temperatures resulted in estimates of median hatching at 258.5, 258.0, and 210.3 ATUs, respectively, which occurred 156.7, 157.3, and 169.6 days after spawning. The corresponding median hatch dates for Arctic char were October 3, 2019, March 16, 2019, and April 21, 2019 (Figure 4). Had lake trout spawned at the same times as Arctic char, their estimated median hatch dates would have ranged from May 18, 2019, to July 5, 2019. Conversely, had Arctic char spawned when lake trout did, their estimated median hatch date would have been January 24, 2019.

### 3.5 Inferring spawning site location of anadromous Arctic char without oviduct tag

**FIGURE 5:**
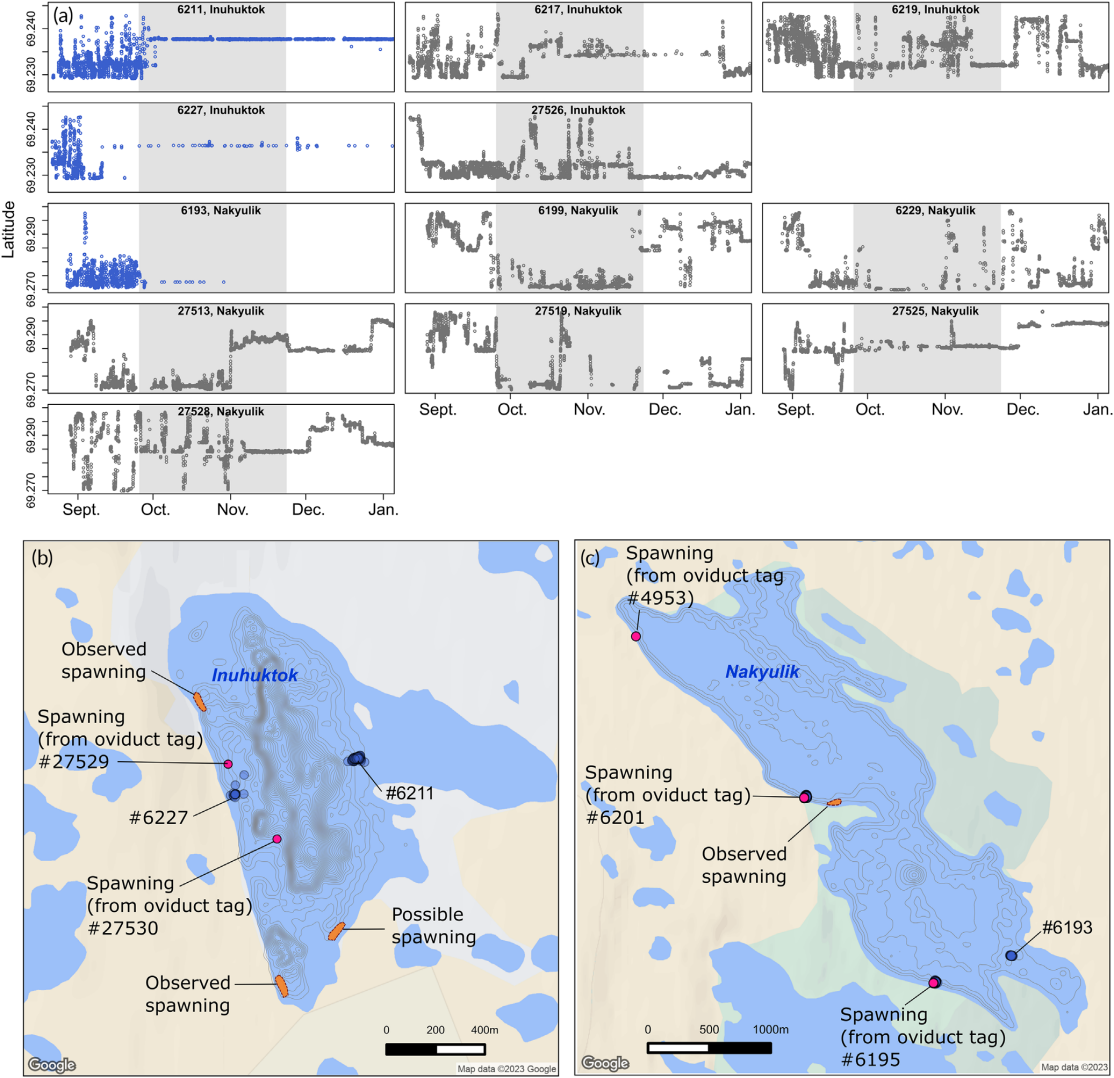
(a) Movements (along latitude) of anadromous Arctic char (*Salvelinus alpinus*) implanted with an abdominal tag but not with an oviduct tag. Sedentary behavior during the presumed spawning period (gray shading) suggests spawning for 6211 and 6227 (Inuhuktok), and 6193 (Nakyulik), shown in blue; (b, c) mapped positions of these inferred spawning fish during a 3‐week period (blue points) and detected spawning locations from oviduct tags (pink points) shown in Figure 2 are also shown for comparison. Areas of spawning observed by Inuit fisher are shown on the maps for comparison (orange polygons). The spawning act was not clearly identified in the “possible spawning” site identified in Inuhuktok, but aggregation of Arctic char in spawning color was suggesting a spawning site.

The spawning behavior and location of anadromous Arctic char implanted with only abdominal tags could be inferred from a prolonged sedentary period. Two Arctic char in Inuhuktok (6211 and 6227) and one Arctic char in Nakyulik (6193) could be identified as probable spawners. The spawning location was also inferred from their sedentary position (Figure 5b,c). In Inuhuktok, one of the inferred spawning sites was in the same area as a spawning site identified from the shedding of an oviduct tag, in shallow (~2 m) water near shore. The other two inferred spawning sites were located on shoals, also in shallow water (<3 m). The three inferred spawners (6211, 6227, and 6193), implanted with sensor tags, used a median depth of, respectively, 1.50, 2.04, and 1.06 m (Figure S9). We could not infer spawning from the remaining nine Arctic char (three in Inuhuktok and six in Nakyulik), as they either did not remain sedentary or were not consistently positioned for at least 3 weeks (Figure 5).

## 4 DISCUSSION

In this study, we used acoustic telemetry transmitters inserted into the oviducts of Arctic char and lake trout, paired with abdominal acoustic transmitters and a fine‐scale acoustic telemetry positioning system, to investigate the timing and location of spawning, as well as the associated behavior. Pairing the oviduct and abdominal tags shed light on the spawning and postspawning behavior of female anadromous Arctic char highlighting that they remained close to the spawning location months after reproduction. This behavior was highly distinct from that exhibited by a lake trout and a resident char (Figure S8).

### 4.1 Technical applicability of oviduct tags to detect spawning in the field

Only 41% of tags (7/17) were shed during presumed spawning events (*n* = 6 for Arctic char and *n* = 1 for lake trout). Our study would have benefited from increased sample sizes, especially given the low percentage of oviduct tags that were correctly expelled. Although this success rate may seem low, it is comparable to previous studies in more southerly locations. For example, in a previous study of muskellunge and northern pike where radio transmitters were inserted into the oviducts, an ejection rate of 50% was inferred (Pierce et al., 2007). Manual stripping of lake trout also implanted with acoustic transmitters led to a higher success rate of 68%, but the stripping continued until tag ejection or all eggs were expelled (Binder et al., 2014). A lack of gonadal maturity or the fact that fish could have skipped reproduction might explain why some fish did not expel the tags in our study, although all tagged char were exhibiting spawning colouration. In muskellunge, large females with ripe eggs were most likely to expel the transmitters (Pierce et al., 2007), an observation that is corroborated by studies in Atlantic salmon (*Salmo salar*) and rainbow trout (*Oncorhynchus mykiss*) (Peaks et al., 1997). In perch, however, a successful oviduct tag expulsion rate of 90% was observed (Skovrind et al., 2013). The fact that Arctic char and lake trout are gymnovarian (eggs are released in the body cavity) whereas perch are cystovarian (continuity between the ovary and the oviduct) might explain this difference. Thus, although the success rate of this approach varies greatly among taxa, we argue that it is a valuable approach for identifying potential spawning grounds of fish in remote locations.

When coupled with an abdominal tag, tag ejection can be better detected by the loss of signal when oviduct tags are presumably ejected in the substrate, as noted by Binder et al. (2014). For species exhibiting typical spawning behavior, the pairing with abdominal tags could also allow spawning to be inferred if oviduct tags are not expelled or expelled prematurely, as evidenced in this study. For anadromous Arctic char, the conjunction of the timing of ejection during the spawning season and a drastic change in behavior allowed us to infer spawning in fish not equipped with oviduct tags.

### 4.2 Insight into fish behavior, spawning habitat, and timing

The spawning female anadromous Arctic char reached their spawning locations and remained sedentary for a few weeks prior to spawning and over the months that followed. They remained close to or on their spawning site during the egg incubation period and, for some of them, possibly until hatching (estimated hatching: March 10 to April 22, 2019). Our observations could indicate that female anadromous Arctic char guard their nests during their prolonged incubation. Indeed, several salmonids are known to show parental care traits after egg fertilization, although for much shorter periods than documented here (McPhee & Quinn, 1998; Tentelier et al., 2011; Van den Berghe & Gross, 1989). Anadromous Arctic char largely cease feeding over winter (Boivin & Power, 1990; Jørgensen & Johnsen, 2014; Moore & Moore, 1974; Young et al., 2021), and remaining sedentary could also reduce energetic costs. Potential nest‐guarding behavior may be beneficial in the study lakes given the presence of other fish that continue feeding throughout winter and could potentially prey on Arctic char eggs or fry. A protective and aggressive behavior exhibited by male anadromous Arctic char that remain close to the nest after spawning has been observed in other locations by Inuit fishers (Dubos, May, et al., 2023) and via snorkeling to observe spawning behavior (Cunjak et al., 1986). Long‐term nest guarding, however, was not observed in stream‐spawning Arctic char over winter in other habitats after spawning (Beddow et al., 1998; Dubos, St‐Hilaire, et al., 2023). For characterization of potential protective behavior by anadromous char, we recommend future studies consider also male behavior relative to the spawning events. Regarding resident Arctic char, given the assumption that they feed over winter, presumably because they accumulate significantly less endogenous energy stores than anadromous char during summer, we could expect them to resume their movements after spawning, which we observed here (27530; Figure S8). In other areas, however, landlocked Arctic char females and males have been observed protecting their eggs (Frye et al., 2021), but only for a few days after spawning.

During the inferred spawning period, the tagged lake trout 6203 (Figure 2) continued to exhibit horizontal movements, with a slightly lower traveled distance 2 days before spawning. Female lake trout are known to move around spawning areas and intermittently lay eggs in multiple suitable habitats (Binder et al., 2021; Esteve et al., 2008). Just after spawning, the tagged female resumed activity, which is consistent with resumed feeding over winter (Blanchfield et al., 2009). Our results, although limited, and those of others (Esteve et al., 2008; Gunn, 1995) suggest that lake trout exhibit limited or no protective behavior of eggs postspawning. Lake trout were also found to be highly mobile during the spawning season by Binder et al. (2018), McMeans et al. (2020), and Blanchfield et al. (2023), and because lake trout did not exhibit marked changes in behavior during the spawning period, the spawning timing and location could not be inferred from abdominal tags alone. The combined use of oviduct and abdominal tags thus offers important advantages for this species.

Throughout and after the spawning season, anadromous Arctic char used a constant shallow depth, close to the inferred spawning location. During fieldwork in summer 2023, two identified spawning locations (27529 in Inuhuktok and 6201 in Nakyulik) were accessed by boat. From the surface, and with the use of underwater cameras, we could observe that both sites exhibited heterogeneous substrate made of cobble (64 to <256 mm), gravel (2 to <64 mm), and a few boulders. The sites were located at a transition zone between the gentle slope of the littoral zone and a steeper drop‐off. During the spring, the earlier melt of the littoral zone allows the inshore water to become productive sooner (Bégin et al., 2020), which could benefit fish in the free‐ and post‐embryo stages. It is unknown at this time if the inferred spawning ground temperatures are locally influenced by underground springs. A recent study has suggested that Arctic fishes may select warmer lake microhabitats for spawning if available (Dubos, St‐Hilaire, et al., 2023). In the present study, the lake trout putatively spawned at a depth of 5.2 m, which is consistent with spawning depths reported for lake trout in southern lakes, although a broad range of depths has been reported (from 0.15 to 91 m, Martin & Olver, 1980; Esteve et al., 2008; Marsden et al., 2016). The use of deeper and warmer waters might be beneficial for lake trout egg incubation at high latitudes, because they require more ATUs than Arctic char for egg development (Dwyer, 1987; Quinn, 2005). Oviduct tags to locate spawning habitats more precisely could be used for more targeted characterization of, for example, substrate, temperature, benthic invertebrates.

We acknowledge that our small sample size might not be fully representative of the temporal window of Arctic char spawning in the region, and some Arctic char probably spawn earlier than our earliest inferred date (end of September). Indeed, according to Inuit knowledge, spawning can be directly observed very early in the fall when the ice is just forming, spawning beds being clearly visible (R. Ekpakohak, personal observation). In addition, it is possible that Arctic char and lake trout use several sites, but even though the use of oviduct tags would not allow to assess all the spawning sites, it would still be valuable to identify the first one or the one where the tag would be ejected. Although hybrids of Arctic char and lake trout have been observed in the Arctic (Hammar et al., 1989; Wilson & Hebert, 1993), the earlier spawning season of lake trout in this system may limit the possibility of hybridization with Arctic char. Despite the small number of presumed spawners, the timing of spawning events in Inuhuktok appeared later than in Nakyulik, which is consistent with observed differences in thermal regimes (Figure 3). In contrast, lake trout are presumed to have spawned in September, during the fall temperature drawdown. As observed during subsequent fieldwork, most (16 of 22) of the adult lake trout (1950 ± 65 g, mean ± SD) caught in the region in late August (August 23–31, 2023) were fully mature, and many were actively spawning or had spawned recently (11 of 22; M. Gilbert, unpublished data). Similarly, Callaghan et al. (2016) also reported lake trout spawning during the first and the last weeks of September in Alexie Lake, Northwest Territories (latitude 62.4°). Lake trout spawning is thought to be associated with declining temperatures, reduced photoperiods, and strong autumn winds, and thus generally occurs earlier at more northern latitudes (Binder et al., 2021; Marsden et al., 2021; Martin & Olver, 1980). At lower latitudes, lake trout reproduction has been documented from October to December, still during a rapid temperature drawdown (Gunn, 1995; Sly & Evans, 1996) or after lake turnover, when the water temperature is below 10°C (Binder et al., 2021; Martin & Olver, 1980). The early spawning of lake trout at high latitudes could be linked to slower embryonic development at cold temperatures (Brannon, 1987; Dwyer, 1987). Indeed, if lake trout in the present study spawned at the same time as Arctic char (October–November) or as temperate lake trout, they could hatch as late as July, limiting their window for summer growth (Quinn, 2005; Skoglund et al., 2012). The predicted hatch timing for Arctic char aligns with direct observations of hatching in the Canadian Arctic (Johnson, 1980; MacCallum & Regier, 1984).

Despite low sample sizes, our study suggests that using paired oviduct and abdominal transmitters facilitates inferences about spawning behavior of other anadromous Arctic char equipped only with abdominal tags, which exhibited similar habitat use and stationary behaviors. In previous studies, spawning is usually validated by recapturing fish or with the previous knowledge of spawning sites to confirm the presence of eggs (e.g., Basilone et al., 2015). Therefore, we argue that our telemetry approach (paired oviduct and abdominal tags on a subset of animals, with other fish receiving only abdominal tags) can likely be used to infer other fish species' spawning, provided they exhibit specific on‐nest behaviors.

### 4.3 Management implications and perspectives

The identification of high‐quality fish spawning habitats is central to their conservation and is a top fisheries research priority in Canada (Dey et al., 2021). Yet spatiotemporal aspects of spawning remain unknown for most Arctic fish populations. Additionally, Arctic aquatic habitats continue to be altered by climate change and other human activities (e.g., Gammons et al., 2009), further stressing the need to identify habitats of critical importance while also providing a baseline for future management initiatives and monitoring. Here, we have highlighted the use of oviduct‐inserted acoustic transmitters as an effective method of identifying spawning locations and timing. Importantly, we also now better understand the behavior of Arctic char around the spawning season, potentially allowing inference of spawning location and timing in future studies in the absence of oviduct‐inserted transmitters.

## 5 CONCLUSION

The use of oviduct tags combined with a fine‐scale telemetry positioning system allowed us to infer the timing and location of spawning for Arctic char and lake trout in two High Arctic lakes. These spawning locations of Arctic char were in the littoral zone close to or in very similar habitat than spawning sites identified by Inuit knowledge holder. Pinpointing the locations of shed oviduct tags using the VPS provided knowledge of otherwise‐cryptic spawning locations and will enable future characterizations of habitats and environmental conditions required for spawning and egg incubation. Pairing abdominal tags with oviduct tags shed light on female anadromous Arctic char behavior before and after spawning, suggesting nest guarding, a behavior not previously documented in this species. We also collected preliminary data suggesting very different spawning behaviors in lake trout in the same lakes.

## AUTHOR CONTRIBUTIONS

Jean‐Sébastien Moore, Richard Ekpakohak, and Les N. Harris designed this study; Les N. Harris, Brendan K. Malley, Richard Ekpakohak, and Matthew J. H. Gilbert conducted the fieldwork. Véronique Dubos and Matthew J. H. Gilbert performed data analysis. Jean‐Sébastien Moore, Les N. Harris, and Nathan B. Furey provided guidance and supervision at various stages of the study. All authors contributed significantly to the writing of the article.

## FUNDING INFORMATION

This study was funded by the Nunavut Wildlife Management Board, Fisheries and Oceans Canada (Nunavut Implementation Funds), and Polar Knowledge Canada Northern Science and Technology grants NST‐1718‐0036 and NST‐2223‐0072. Véronique Dubos and Matthew J. H. Gilbert were funded by the Weston Family Foundation Fellowships for Northern Research. Matthew J. H. Gilbert was also supported by NSERC and ArcticNet‐MEOPAR postdoctoral fellowships. Nathan B. Furey was supported by the Class of 1937 Professorship in Marine Biology by UNH's School of Marine Science and Ocean Engineering.

## Supporting information


**Data S1.** Supporting information.
