# Supplementary material for: The potential of oviduct tags and fine‐scale acoustic telemetry to reveal the timing and location of spawning in Arctic salmonids (Salvelinus spp.)
Source: J Fish Biol. 2024 Oct 7;106(5):1639–53. doi: 10.1111/jfb.15951 (PMC12120342; doi:10.1111/jfb.15951)
Supplement: Supplementary file 1 — Data S1. Supporting information. [file JFB-106-1639-s001.docx]

**Supporting information for**

**Oviduct tags and fine-scale acoustic telemetry can reveal timing and location of spawning in two Arctic salmonids (*Salvelinus* spp.)**

Véronique Dubos, Institut de Biologie Intégrative et des Systèmes and Département de Biologie, Université Laval, Québec, Canada

Les N. Harris, Fisheries and Oceans Canada, Arctic and Aquatic Research Division, Winnipeg, Canada

Richard Ekpakohak, Elder and Cambridge Bay Resident, Cambridge Bay, Canada

Brendan K. Malley, Fisheries and Oceans Canada, Arctic and Aquatic Research Division, Winnipeg, Canada

Matthew J.H. Gilbert*, Department of Biology, University of New Brunswick, Saint John, NB, Canada

Nathan B. Furey, Department of Biological Sciences, University of New Hampshire, Durham, NH, USA

Jean-Sébastien Moore, Institut de Biologie Intégrative et des Systèmes and Département de Biologie, Université Laval, Québec, Canada

*Matthew J.H. Gilbert current affiliation is Institute of Arctic Biology and Department of Biology and Wildlife, University of Alaska, Fairbanks, AK, USA

**Table S1. List of tag in lakes Inuhuktok and Nakyulik. When applicable, the paired oviduct tag (V7) is indicated in parenthesis. Oviduct tags presumably ejected during spawning are highlighted in blue. Asterisk (*) indicates anadromous Arctic char and ** indicates resident Arctic char, confirmed by detections in the same lakes in the summer of subsequent years and no detection outside their respective lakes.**

| **Inuhuktok, Arctic char** | | | | |
| --- | --- | --- | --- | --- |
| **Tag** | **Sex** | **Fork length (mm)** | **Weight (g)** |  |
| 6211, V16 TP* | F | 730 | 4350 |  |
| 6219, V16 TP* | M | 738 | 4600 |  |
| 6217, V16 TP* | M | 748 | 4100 |  |
| 6227, V16 TP (Oviduct 4962)* | F | 718 | 4100 |  |
| 27512, V16** | M | 557 | 1900 |  |
| 27526, V16* | M | 558 | 1800 |  |
| 27529, V16 (Oviduct 4947)* | F | 686 | 3400 |  |
| 27530, V16 (Oviduct 4949)** | F | 670 | 3300 |  |

| **Inuhuktok, Lake trout** | | | | |
| --- | --- | --- | --- | --- |
| **Tag** | **Sex** | **Fork length (mm)** | **Weight (g)** |  |
| 6205, V16 TP | M | 1150 | 13000 |  |
| 6207, V16 TP | M | 562 | 2000 |  |
| 6215, V16 TP | M | 536 | 1800 |  |
| 6225, V16 TP | F | 618 | 2800 |  |
| 27517, V16 | F | 556 | 2200 |  |
| 27520, V16 | F | 516 | 1700 |  |
| 27523, V16 | M | 535 | 1550 |  |
| 27531, V16 | F | 1005 | 6700 |  |

| **Nakyulik, Arctic char** | | | | |
| --- | --- | --- | --- | --- |
| **Tag** | **Sex** | **Fork length (mm)** | **Weight (g)** |  |
| 4948, V7 | M | 539 | NA |  |
| 4952, V7 | F | 475 | 1300 |  |
| 4953, V7 | NA | NA | 1300 |  |
| 4959, V7 | F | 490 | NA |  |
| 4961, V7 | F | 610 | NA |  |
| 4965, V7 | F | 600 | NA |  |
| 6193, V16 TP* | M | 564 | 1800 |  |
| 6195, V16 TP (Oviduct 4957)* | F | 542 | 1800 |  |
| 6199, V16 TP* | M | 790 | 5200 |  |
| 6201, V16 TP (Oviduct 4956)* | F | 604 | 2500 |  |
| 6229, V16 TP* | M | 730 | 4750 |  |
| 6231, V16 TP (Oviduct 4954) | F | 661 | 3350 |  |
| 27513, V16* | M | 548 | 1850 |  |
| 27516, V16 ** (Oviduct 4950) | F | 558 | 2000 |  |
| 27519, V16* | M | 551 | 1900 |  |
| 27522, V16** | NA | NA | 2050 |  |
| 27525, V16* | M | 584 | 2300 |  |
| 27528, V16* | M | 598 | 2300 |  |

| **Nakyulik, Lake trout** | | | |
| --- | --- | --- | --- |
| **Tag** | **Sex** | **Fork length (mm)** | **Weight (g)** |
| 4960, V7 | F | 1000 | NA |
| 6197, V16 TP (Oviduct 4958) | F | 772 | 5400 |
| 6203, V16 TP (Oviduct 4964) | F | 526 | 2100 |
| 6209, V16 TP | M | 565 | 2250 |
| 6213, V16 TP | F | 848 | 6200 |
| 6221, V16 TP | M | 556 | 1900 |
| 6223, V16 TP | F | 1010 | 18000 |
| 27514, V16 | F | 636 | 2600 |
| 27515, V16 | M | 745 | 4400 |
| 27518, V16 | M | 635 | 2700 |
| 27521, V16 | M | 570 | 2000 |
| 27524, V16 | M | 602 | 2250 |
| 27527, V16 (Oviduct 4951) | F | 576 | 2250 |

**
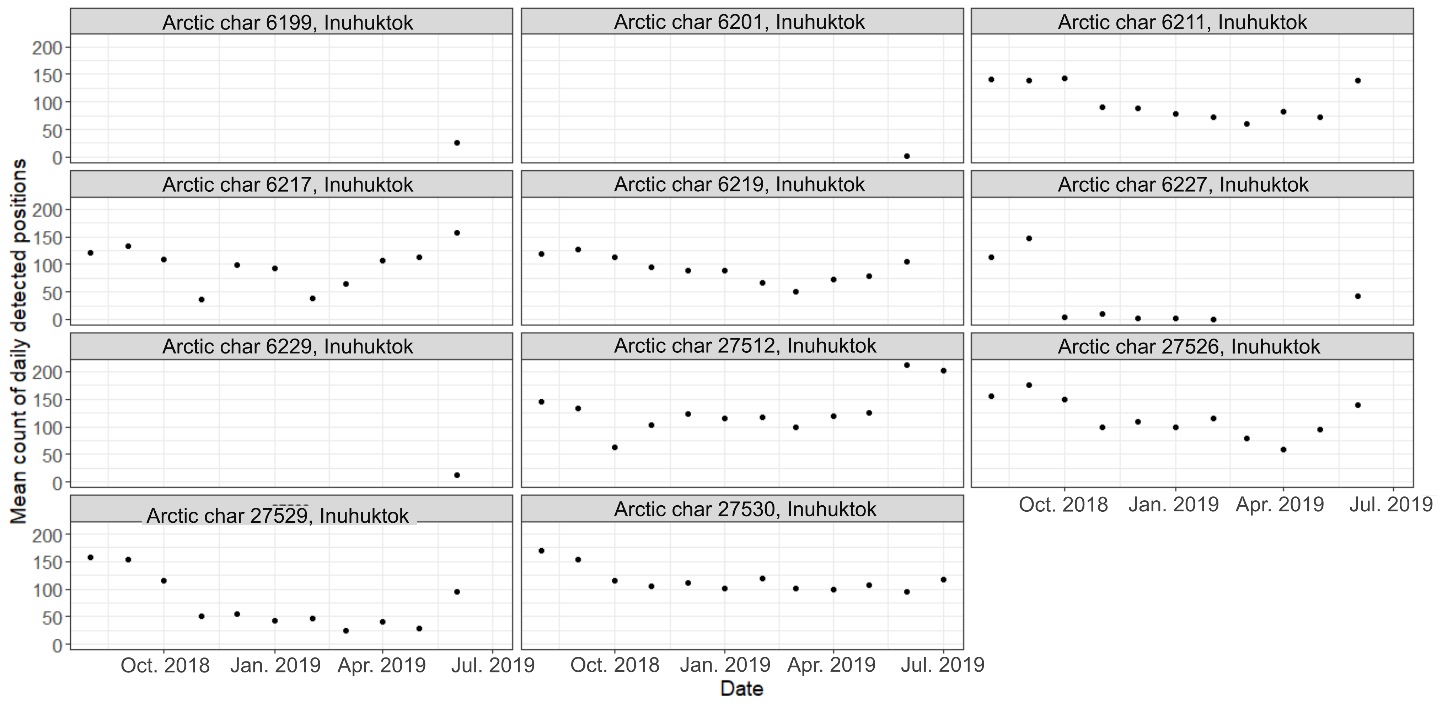
**

**Figure S1. Mean daily calculated VPS positions for Arctic char in Inuhuktok. Arctic char #6199, 6201 and #6229 are from Nakyulik Lake and were detected in Inuhuktok in June 2019 during their downstream migration.**

**
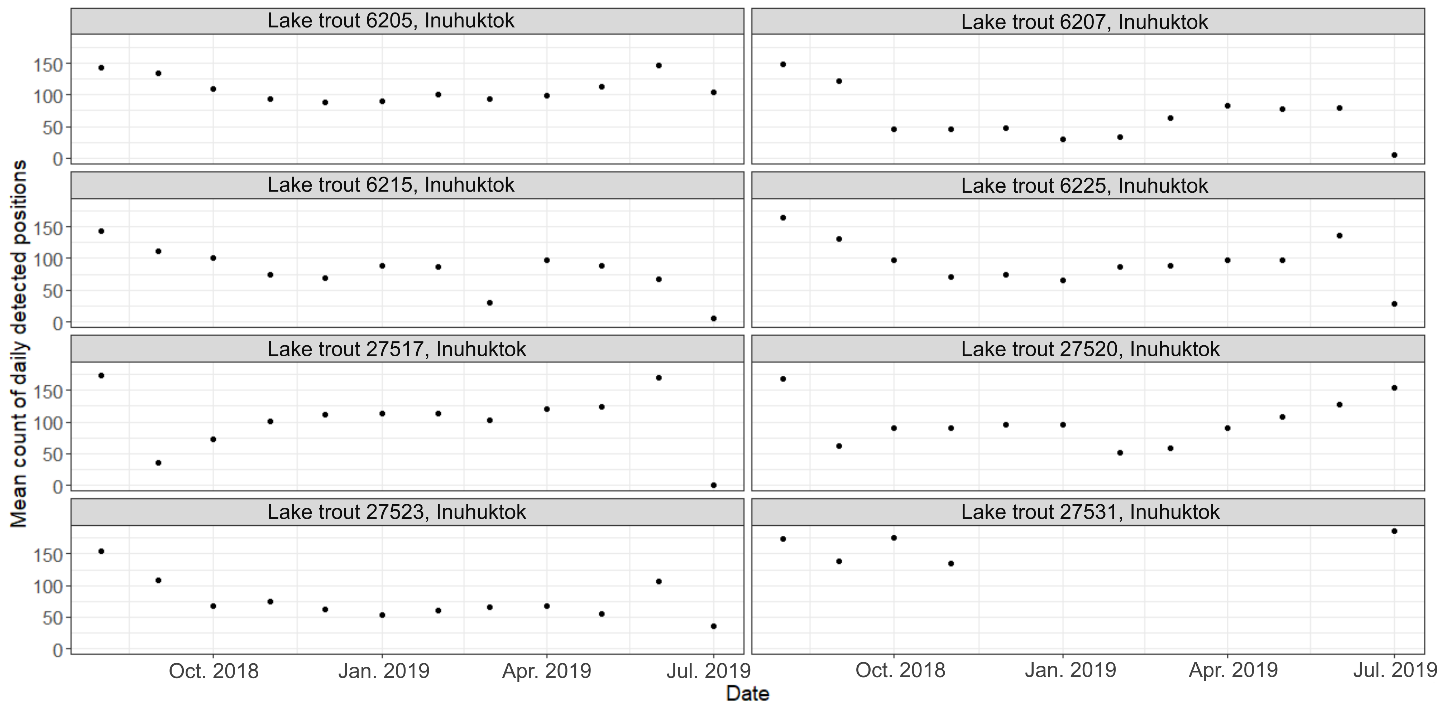
**

**Figure S2. Mean daily calculated VPS positions for lake trout in Inuhuktok.**

**
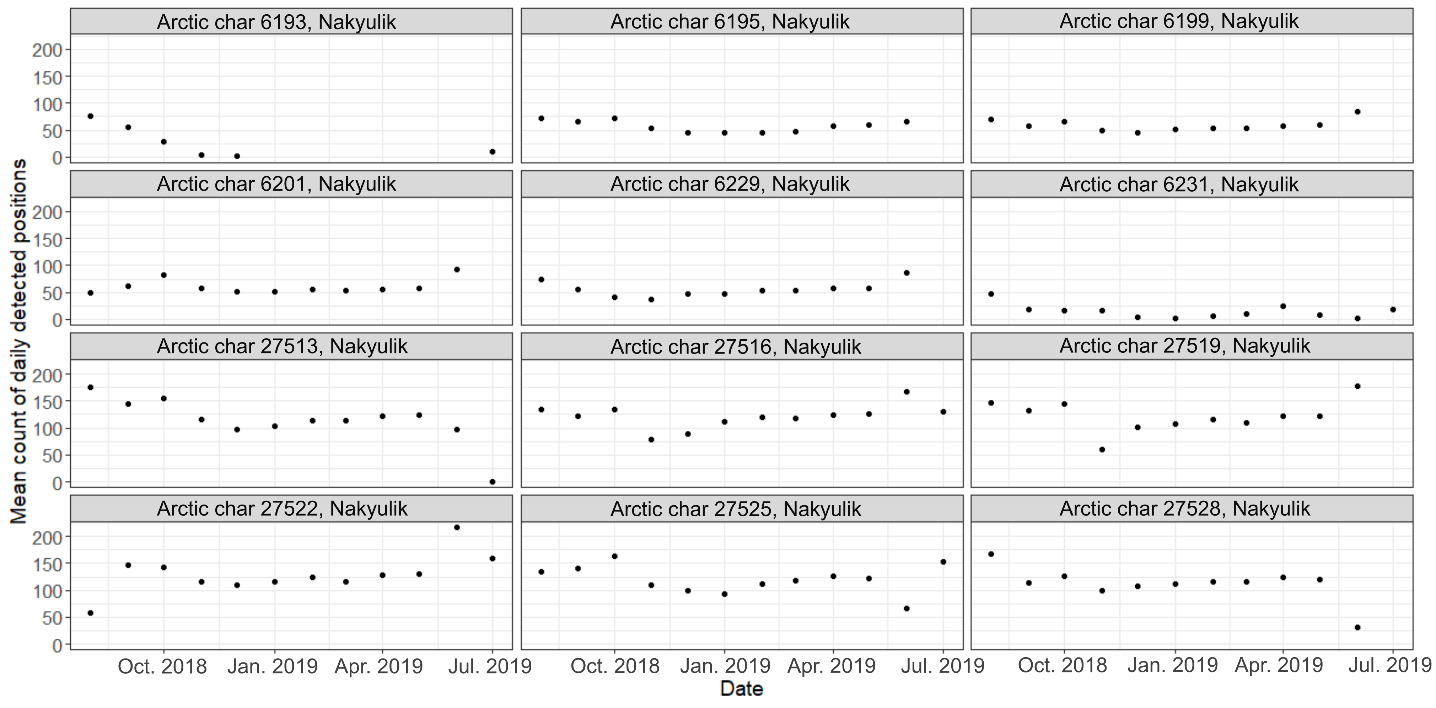
**

**Figure S3. Mean daily calculated VPS positions for Arctic char in Nakyulik.**

**
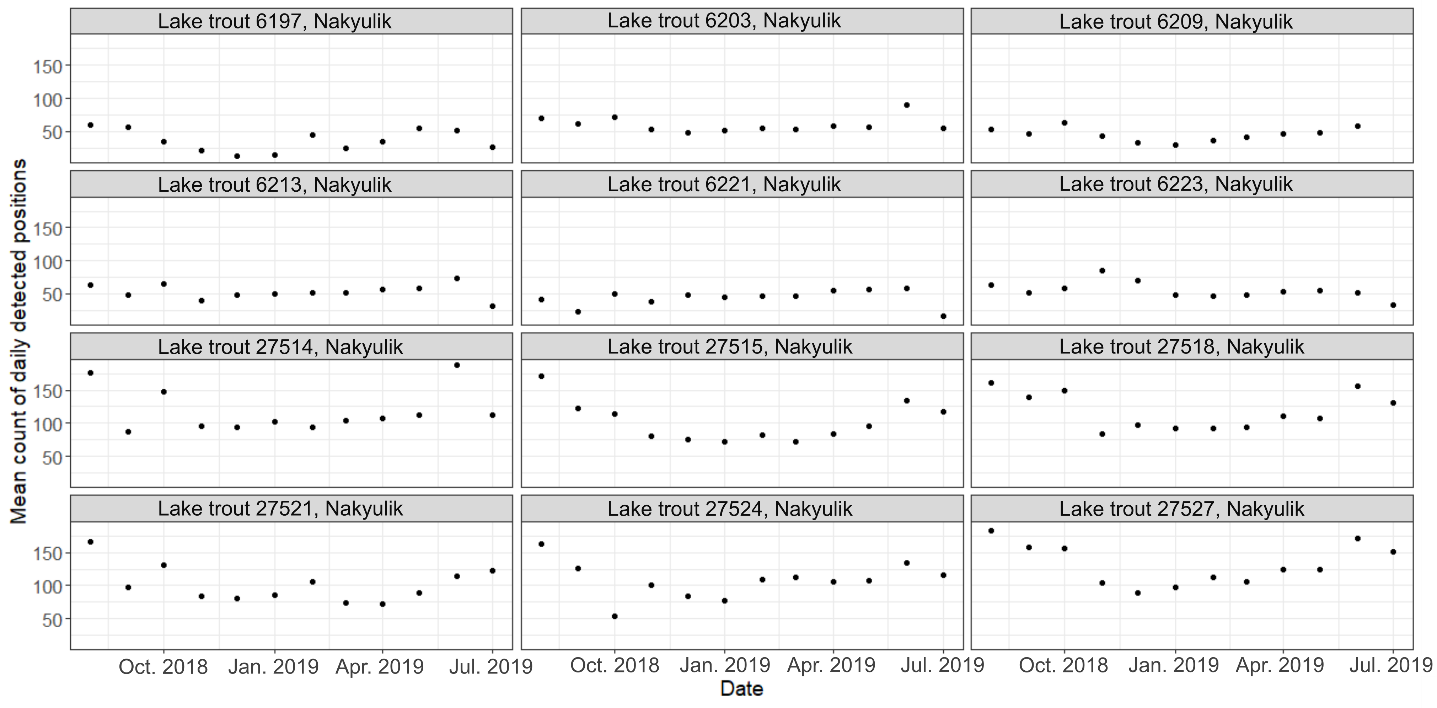
**

**Figure S4. Mean daily calculated VPS positions for lake trout in Nakyulik.**

**
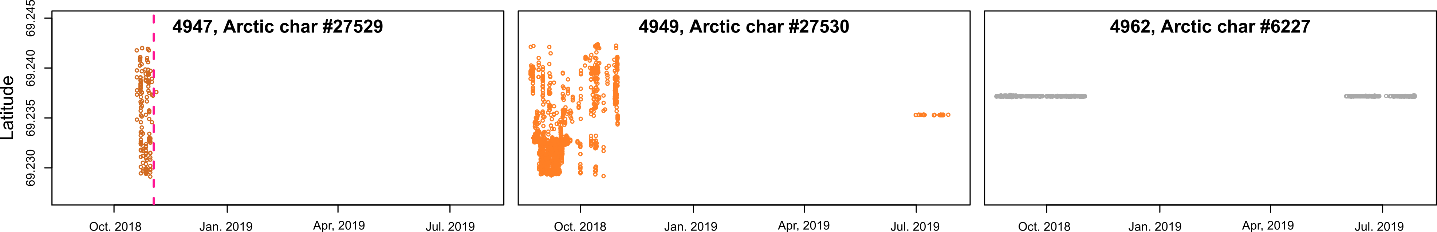
Figure S5. Arctic char oviduct tag movements (according to latitude) in Inuhuktok Lake. Orange tags are inferred as ejected during the spawning. The grey tag was lost prematurely. The dashed vertical line identifies the tag ejection date.**


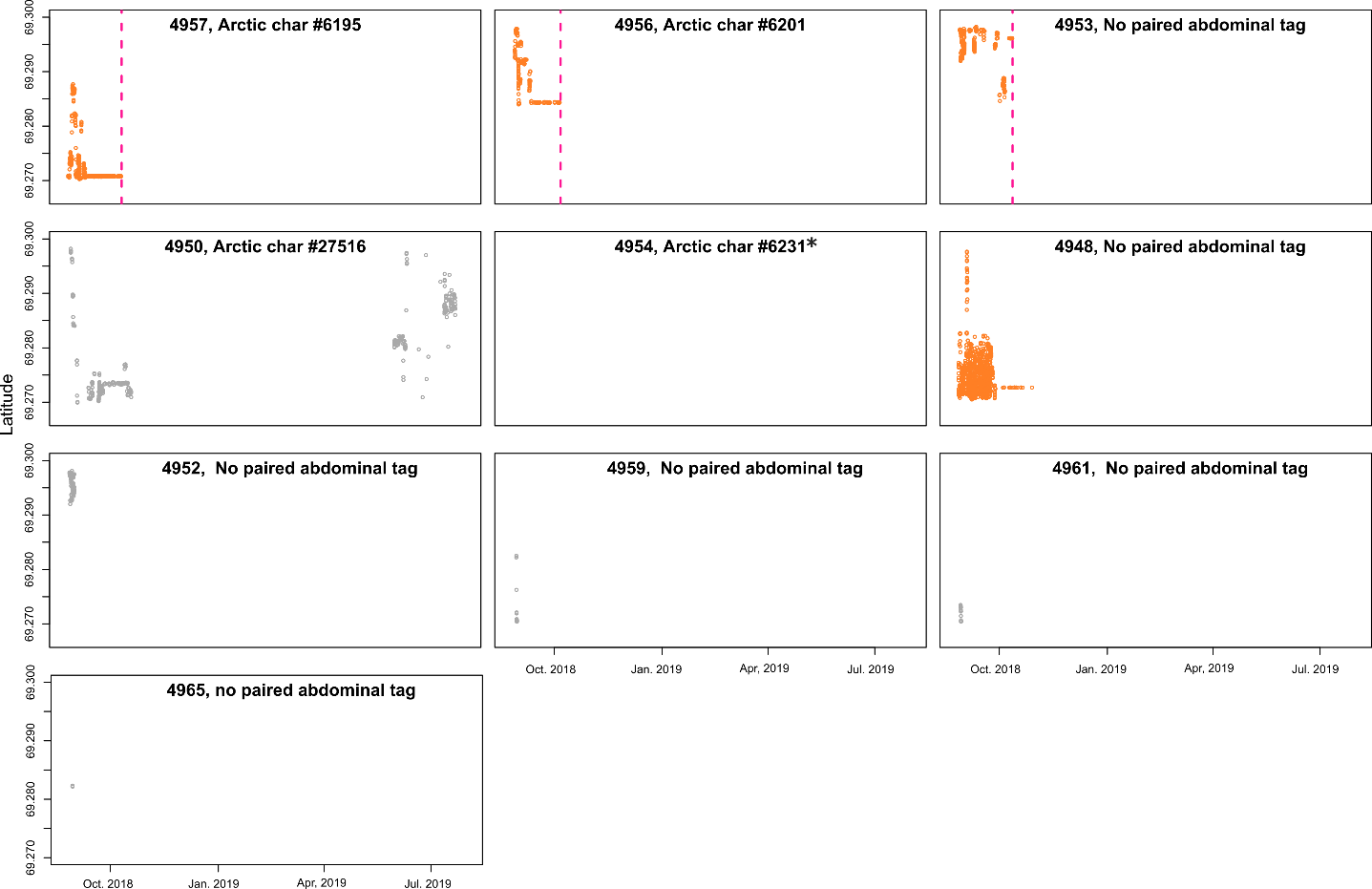


**Figure S6. Arctic char oviduct tag movements (according to latitude) in Nakyulik Lake. Orange tags are inferred as ejected during the spawning. The grey tags were either lost prematurely or not ejected during the spawning period. The dashed vertical line identifies the tag ejection date. Oviduct tag 4954 was prematurely ejected after 4 detections, which don’t show on the plot because the HPE of these positions were all >14. Oviduct tag 4953 was likely ejected during a spawning event after Nov 1^st^ and the spawning timing could not be established.**


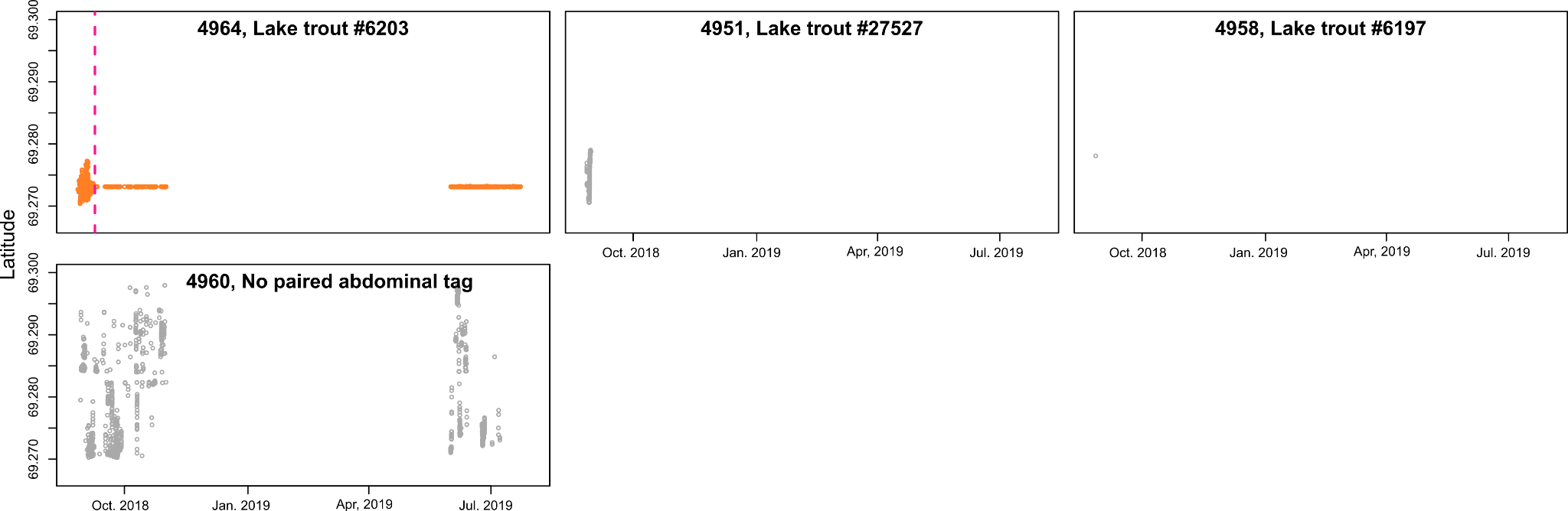


**Figure S7. Lake trout oviduct tag movements (according to latitude) for lake trout in Nakyulik Lake. Orange tag is inferred as ejected during the spawning. The grey tags were either lost prematurely or not ejected during the spawning period. The dashed vertical line identifies the tag ejection date.**


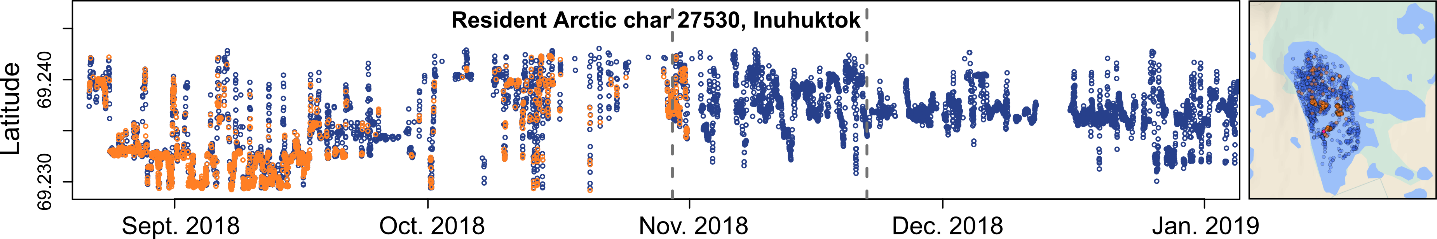


**Figure S8. Movements of a resident Arctic char (blue) with oviduct tag (orange). The oviduct tag (#4949) was ejected after 2018-11-01, while the tag was programmed to stop transmitting, preventing us from determining the spawning timing. The spawning site could however, be located as tag resumed transmitting on June 1^st^ 2019 (see Figure S1, middle panel). The abdominal tag showed that the resident Arctic char did not remain at the spawning site and continued to travel in the lake with a similar level of activity as prior to spawning, contrary to the anadromous Arctic char.**

**
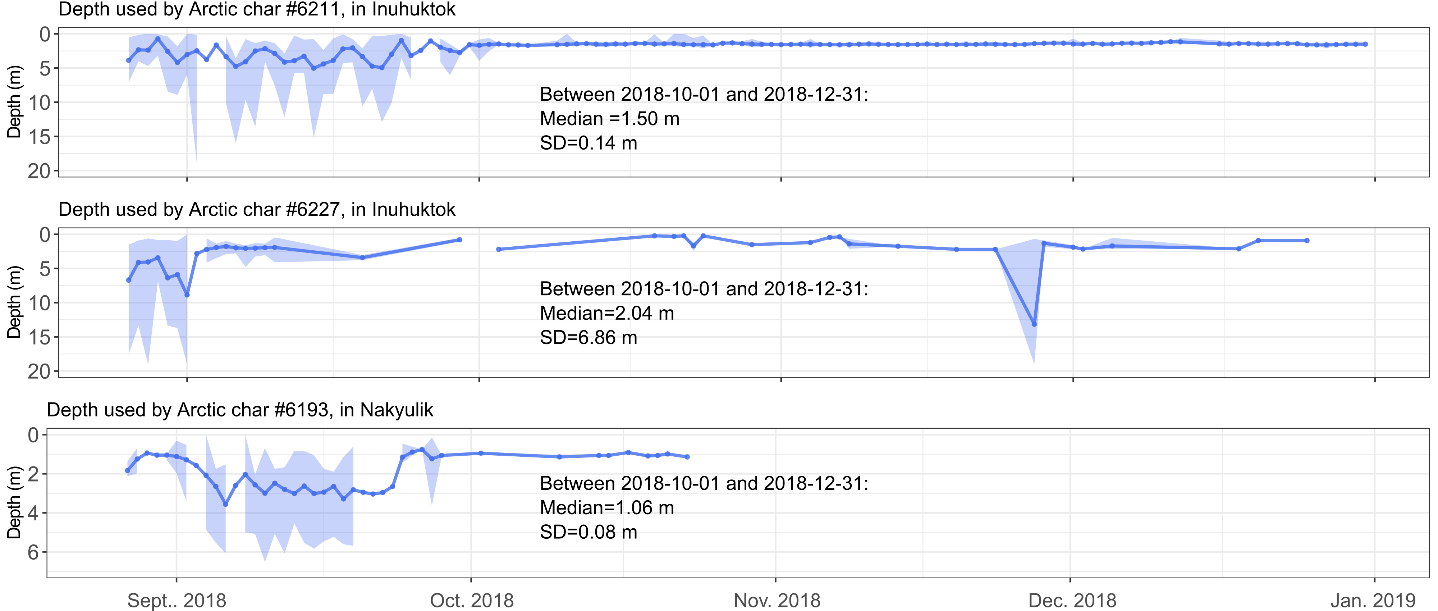
**

**Figure S9. Daily mean depth used by the inferred spawning Arctic char equipped with sensor tags. The shaded areas illustrate the minimum and maximum daily values recorded.**
